# Validation of the questionnaire of olfactory disorders (QOD) for the Brazilian population

**DOI:** 10.1016/j.clinsp.2024.100414

**Published:** 2024-06-14

**Authors:** Amanda Beatriz Costa da Silva Bernardino, Márcio Andrade Barreto-Filho, Amanda Savieto Pompeu, Jaqueline dos Santos Andrade, Amanda Canário Andrade Azevedo, Michelle Queiroz Aguiar Brasil, Mariana Cedro, Cintia Araújo, Nilvano Andrade, Kevan Akrami, Henrique Ochoa Scussiatto, Viviane Sampaio Boaventura, Marco Aurélio Fornazieri

**Affiliations:** a Health Sciences Center of the Universidade de Londrina, Londrina, PR, Brazil; b Instituto Gonçalo Moniz, Fundação Oswaldo Cruz, Salvador, BA, Brazil; c Universidade Federal da Bahia, Salvador, BA, Brazil; d Section of Otorhinolaryngology at Hospital Santa Izabel, Salvador, BA, Brazil; e University of California, San Diego, Division of Infectious Disease, Department of Medicine, San Diego, California, USA; f Department of Surgery, University of Illinois - Chicago, Chicago, Illinois, USA; g Pontifícia Universidade Católica do Paraná, Londrina, Paraná, Brazil; h Smell and Taste Center, Department of Otorhinolaryngology Head and Neck Surgery, Perelman School of Medicine, University of Pennsylvania, Philadelphia, Pennsylvania, USA

**Keywords:** Olfaction disorders, Quality of life, Survey and questionnaires, Validation study

## Abstract

**Background:**

The incidence of olfactory disorders has increased in recent years, mainly related to COVID-19 infection. In Brazil, over 37 million cases of COVID-19 have been reported, and approximately 10 % of those cases continue to experience olfactory disorders for more than one month. Despite the significant negative impact on well-being, there is currently no validated instrument to assess how olfactory disorders impact the quality of life in Brazil.

**Objectives:**

This study aimed to validate the Questionnaire of Olfactory Disorders (QOD) for Brazilian Portuguese.

**Methods:**

The authors first performed translation, back-translation, expert review, pre-testing, psychometric evaluation and cultural adaptation of the English version of the questionnaire. To assure linguistic and conceptual equivalence of the translated questionnaire, 126 participants from two Brazilian states and varying degrees of olfactory loss answered the QOD and the World Health Organization Quality of Life bref (WHOQOL-bref) questionnaires. The University of Pennsylvania Smell Identification Test (UPSIT®) was used to quantify the olfactory loss. Furthermore, to evaluate the reliability of the Portuguese version a test-retest was performed on a subgroup of patients. The authors observed a high Cronbach's alpha (α = 0.86) for internal consistency of the quality of Life (QOD-QOL) statements.

**Findings:**

As expected, there was a negative correlation between QOD-QOL and UPSIT® (Spearman's ρ = -0.275, *p* = 0.002), since QOL score increases and UPSIT® score decreases with worsening of olfactory function. Correlations were moderate between QOD-QOL and WHOQOL-bref mean (Spearman's ρ = -0.374, *p* < 0.001) and weak to moderate between the QOD-QOL and Visual Analog Scale of the QOD regarding professional life, leisure, and private life (Spearman's ρ = -0.316, *p* = 0.000; Spearman's ρ = -0.293, *p* = 0.001; Spearman's ρ = -0.261, *p* = 0.004; respectively).

**Conclusion:**

In conclusion, the authors have demonstrated a high internal consistency and validity of the Brazilian Portuguese version of the QOD for evaluating the quality of life in individuals with olfactory disorders.

## Highlights

• Olfactory loss correlates with lower quality of life.
• QOD is a reliable tool for olfactory assessment in Brazil.
• QOD validation fills a crucial gap in Brazilian olfactory research.

## Introduction

Olfactory Dysfunction (OD) affects a significant portion of the population, with an estimated prevalence of 4.5 %, which increases with age.^1^ Its impact extends beyond an impaired sensory experience alone, with several negative outcomes including depression and feelings of loneliness,^2^ lack of motivation and sexual dysfunction,^3^ and reduced overall quality of life.^4^ OD can be triggered by upper respiratory tract infections, acute and chronic rhinosinusitis, traumatic brain injury, exposure to toxic substances^5^, ^6^, ^7^, ^8^, ^9^, ^10^, ^11^ and neurodegenerative diseases such as Parkinson's disease^12^ and Alzheimer's dementia.^13^ Recently, a heightened prevalence of OD has emerged during the COVID-19 pandemic, with 10 % of patients presenting with some degree of alteration 6 months after their acute infection.^14^

Despite the high prevalence of olfactory dysfunction, the extent of dysfunction is still underestimated.^15^ As such, accurate instruments are required to assess how olfactory loss affects Quality of Life. Questionnaires used for this purpose are divided into general and specific groups. Questionnaires such as the World Health Organization Quality of Life Questionnaire (WHOQOL-bref),^16^ McGill Quality of Life Questionnaire,^17^ Short-Form Health Survey (SF-36)^18^ are examples of surveys that measure the overall quality of life and can be used for individuals with olfactory loss,^19^ as well as for individuals with other conditions. However, to our knowledge, the only specific questionnaire to evaluate the quality of life in individuals with olfactory loss is the Questionnaire of Olfactory Disorders (QOD).^20^

However, QOD adaptation and validation have yet to be performed in a Brazilian population. There is a need for an instrument capable of measuring the quality of life in individuals with olfactory dysfunction in diverse populations such as Brazil. Therefore, the aim of the present study is the linguistic and cultural validation of the QOD in a Brazilian population.

## Materials and methods

**Fig. 1:**
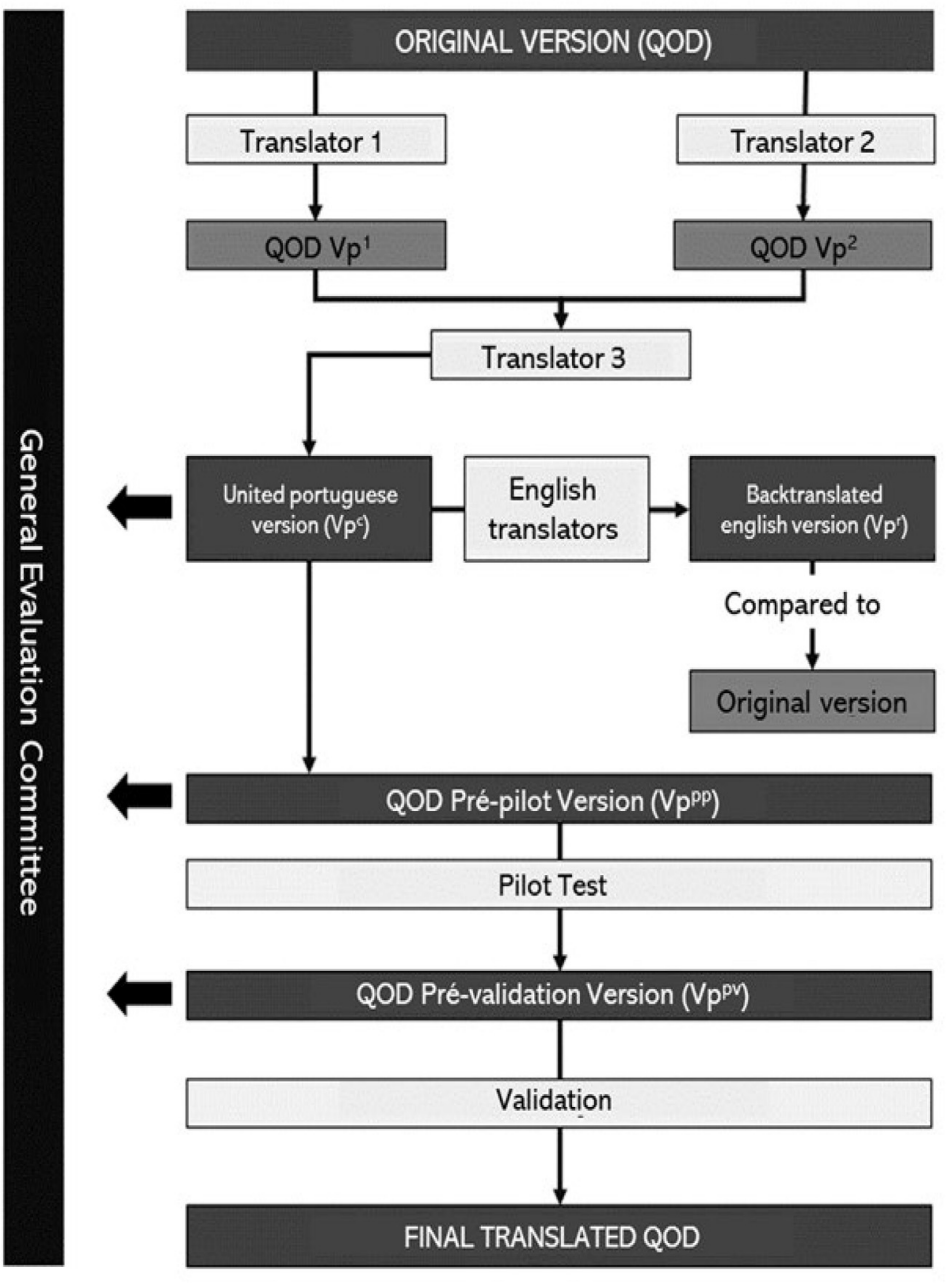
Study design. QOD, Questionnaire of Olfactory Disorders.

The process of validation of the Questionnaire of Olfactory Disorders to Brazilian Portuguese is summarized in Fig. 1.

### Population

One hundred and twenty-six adults were recruited to participate in the study between May 2018 and August 2022, distributed across two centers. Sociodemographic and clinical data were obtained through questionnaires administered by trained interviewers. Olfactory measurement was performed using the University of Pennsylvania Smell Identification Test (UPSIT®),^21^ and quality of life data were obtained by applying the WHOQOL-bref[16] and QOD.^20^ Patients between 18 and 65 years old with complaints of post-infectious olfactory dysfunction from allergic rhinitis, chronic rhinosinusitis with or without polyps, post-traumatic or idiopathic etiology were included. Participants with incomplete questionnaires, olfactory dysfunction not documented by psychophysical testing, olfactory loss due to multiple etiologies, cognitive deficits, age greater than 65 or less than 18 years old, and pregnant women were excluded.

The authors conducted a test-retest questionnaire with 12 patients to assess the reliability of the Portuguese version of the QOD. The retest, including only the QOD and UPSIT®, was applied in Londrina, with an average time span of 4.06 months (3.2‒5.3) between the two applications.

All participants signed an informed consent form. This study was conducted with the approval of the Ethics Committee of the State University of Londrina (UNEL, protocol n° 48,238,421.9.0000.5231), the State University of Bahia (UNEB, protocol n° 38,281,720.2.0000.0057), and the Santo Antônio Hospital (OSID, protocol n° 33,366,030.5.0000.0047).

### Translation and re-translation

The Beaton Cross-cultural translation and validation guideline was used to guide the process of cultural translation.^22^ The original English Version (Table S1) was translated into Portuguese by two native Brazilian translators: an otorhinolaryngology specialist and a non-specialist translator. Each translation resulted in two versions of the questionnaire in Portuguese. A third non-specialist and non-study-related translator was responsible for unifying the two versions, producing a combined version of the questionnaire. To evaluate the translation steps, a committee was created consisting of principal researchers, an otorhinolaryngology specialist, a psychologist, and two non-specialists.

The final version was back-translated into English by two independent translators, a specialist and a non-specialist, both fluent in English. The committee compared the back-translated version with the original version and made semantic changes that, after approval by the Brazilian translators, resulted in the pilot version of the test (Table S2). The QOD pilot was answered by 30 Portuguese speakers, and in each application, they were asked for their understanding of the statements, degree of relevance, and suggestions about its construction. With the collected data, the first version of the pilot test was re-evaluated by the committee and approved for the validation stage. The study design of the validation process can be found in Fig. 1.

### The olfactory disorders questionnaire (QOD)

The QOD was developed to qualitatively assess the degree of olfactory dysfunction during daily life activities. Originally, it consisted of 52 items divided into three domains: negative statements, positive statements, and socially desirable statements. However, the internal consistency of the questionnaire was unsatisfactory (α = 0.54), leading to a decision to reduce the questionnaire.^23^ In this study, the authors used the questionnaire, developed by the author, composed of 29 statements that can be divided into three domains: Quality of Life (QOL), consisting of 19 questions to assess the impact of olfactory disorders on daily life; Sincerity (S), consisting of 6 questions that evaluate the tendency of socially adequate responses; and Parosmia statements (P), 4 questions that measure the existence and degree of parosmia. Each question is answered on the following scale: “I totally agree”, “Partially agree”, “Partially disagree” and “Totally disagree”.

Each domain's score is evaluated independently by summing each question's punctuation. Patients' answers are ordinarily translated with “totally disagree” is equivalent to 0 points and “totally agree”, to 3 points. In order to avoid automatic responses, all domains present negative statements, which are scored in reverse.

The domains' interpretation consists in comparing the raw total score with the maximum value for each stratum (57 points for LQ; 18 points for S; 12 points for P). High LQ, S and p-values indicate strong life quality impairment, the tendency towards giving socially desired answers and parosmia, respectively.

In addition, the QOD includes five Visual Analog Scale (VAS) questions indicating how much olfactory loss impacts different aspects of a patient's life, such as work, leisure, and personal life. All five VAS questions are continuously scored on a 0‒100 scale and interpreted independently.

Additional information regarding questionnaire scoring and interpretation is available in the supplementary materials, which include the complete application instructions for the QOD.

### Assessment of overall quality of life using the WHOQOL-bref

The WHOQOL-bref is a rapid self-administered test used to assess the global quality of life. It was developed by the World Health Organization Quality of Life Group as a short version of the WHOQOL-100.^16^ The WHOQOL-bref has 26 questions, 2 of which are general and 24 are related to specific domains, i.e., physical health, psychological, social relationships, and environment (Fig. S1).

The characteristics of the physical health domain are related to the presence of pain and discomfort, fatigue, and lack of mobility in daily activities. The psychological domain has questions related to thoughts, memory, concentration, and self-esteem. The social relationships domain is related to personal relationships, social support, and sexual activity. The environment domain has questions associated with freedom, physical safety and protection, financial resources of the household environment, health and social care, accessibility and quality of opportunities for acquiring new information and skills, participation and opportunities for recreational/leisure activities, physical environment (pollution/noise/traffic/climate), and transport.^16^ Higher mean scores are related to higher quality of life.^16^

### Psychophysical olfactory function measurement

The psychophysical olfactory function was measured by applying the UPSIT® questionnaire, a self-administered test.^21^ The UPSIT® consists of four booklets, ten pages, and forty questions about odorants, each question has four options. Each page contains a microencapsulated odorant, which the participant must scratch in the indicated area to release the odor. After that, the interviewee smells the odor released and answers the corresponding question. If the participant answers the question correctly, one point is added to the final score, which can range from 0 to 40. Individual scores are used to classify patients as normosmia, mild hyposmia, moderate hyposmia, severe hyposmia, or anosmia based on the score, sex, and age of the patient.^24^

### Statistical analysis

All data were collected, organized, and verified using the REDCap platform (“Research Electronic Data Capture”) and analyzed by the SPSS version 17.0 (Statistical Packages for the Social Sciences, SPSS Inc., Chicago, Illinois, USA). Graphs were created using GraphPad Prism 6 (GraphPad Software Inc., San Diego, CA, USA). Qualitative results were described using absolute (n) and relative (%) frequencies. The normality of the sample was tested by analyzing numerical parameters (mean, median, standard deviation, skewness, and kurtosis), graphs (histogram analysis), and statistical tests (Shapiro-Wilk and Kolmogorov-Smirnov). Quantitative results were described using mean (± standard deviation) or median (interquartile range), according to normality. A Cronbach's alpha coefficient above 0.7 was considered sufficient to evaluate the internal consistency of the QOD. The split-half method was used to evaluate reliability. The ANOVA test was used to evaluate QOD scores among UPSIT® classifications (normosmia, hyposmia, and anosmia). Spearman's correlation was used to compare QOD domains and WHOQOL-bref scores and raw UPSIT® scores. For the description of the frequency of statements, “I totally agree” and “Partially agree” were considered positive responses; p-values < 0.05 were considered statistically significant.

## Results

Between May 2018 and August 2022, 126 patients participated in the validation phase of the study (N Salvador-BA = 23; N Londrina-PR = 103). The majority were women (*n* = 81, 63 %), 40 (+14) years-mean, 58 % presenting severe olfactory loss or anosmic at the time of first evaluation. Post-infectious causes were the most prevalent presumed etiology for olfactory loss (*n* = 73, 57.9 %), regardless of the evaluation center. All 23 patients evaluated in Salvador-BA (CPC) presented with recent olfactory loss associated with post-acute COVID-19 infection. Notably, patients from CPC were older, less educated, and presented with severe olfactory dysfunction (UPSIT), Worse general Quality of Life (WHOQOL) and olfactory-associated QoL (QOD) scores, when compared to Londrina-PR center. Baseline characteristics and descriptive data between the populations of Salvador and Londrina can be found in Table 1.

**Table 1:** Sociodemographic and clinical characteristics of the population divided by application center.

Variable	Total (*n* = 126)	Salvador-BA (*n* = 23)	Londrina-PR (*n* = 103)
Age (years), mean (sd)	40 (14.2)	51 (7.2)	37.6 (14.2)
Female sex-n (%)n (%)	81 (64.3)	17 (73.9)	64 (62.1)
College Educated-n (%)	75 (59.5)	5 (21.7)	70 (68,0)
Smoking habits, n (%)	13 (10.3)	0 (0)	13 (12.6)
**Etiologies, n (%)**			
Post-infectious	73 (57.9)	23 (100)	50 (48.5)
Chronic rhinosinusitis	27 (21.4)	‒	27 (26.2)
Others (idiopathic and traumatic)	26 (20.9)		26 (25.3)
**Olfactory status according to UPSIT®, n (%)**			
Mild loss	24 (19.0)	2 (8.7)	22 (21.4)
Moderate loss	29 (23.0)	4 (17.4)	25 (24.3)
Severe loss	45 (35.7)	12 (52.2)	33 (32)
Anosmia	28 (22.2)	5 (21.7)	23 (22.3)
**Qualitative alterations of sense of smell, n (%)**			
Parosmia	47 (37.3)	10 (43.5)	37 (35.9)
Phantosmia	32 (25.4)	5 (21.7)	27 (26.2)
**QOD, median (IQR)**			
Sincerity	6 (4‒9)	11 (6‒14)	6 (4‒8)
Quality of life	20 (12‒31)	31 (21‒39)	19 (11‒29)
Parosmia	5 (2‒8)	8 (4‒10)	4 (2‒7)
**WHOQOL-bref, mean (SD)**			
General score	3.8 (0.7)	3.1 (0.7)	3.8 (0,6)
Physical	3.6 (0.7)	3.0 (0.8)	3.7 (0.6)
Psychological	3.8 (1.3)	3.1 (0.8)	3.9 (1.3)
Social	3.8 (0.8)	3.2 (1.0)	3.9 (0.7)
Environment	3.8 (0.6)	3.0 (0.8)	4.0 (0.5)

The following data were described as n (%) unless otherwise specified.

**Fig. 2:**
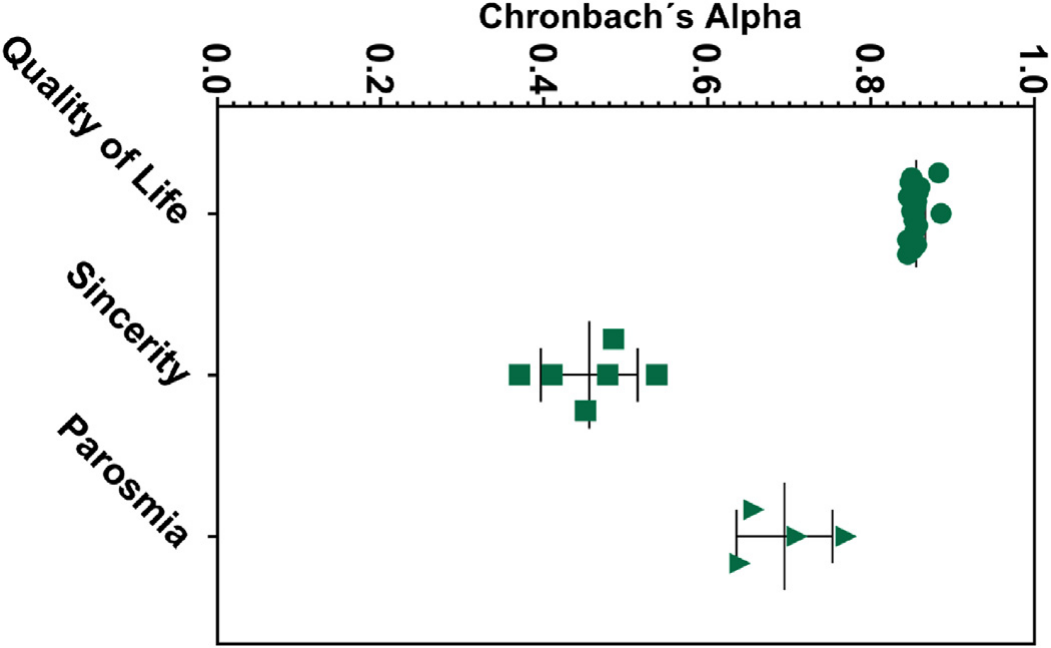
Scatter plot of Cronbach's alpha values for QOD domains. Each data point represents a different alpha coefficient obtained by excluding a single item and calculating the Cronbach's coefficient independently.

Cronbach's α was used for QOD internal consistency evaluation, obtaining a reasonable reliability coefficient (α = 0.86) for the QoL domain (Table 2). The values remained higher than 0.84 with item-item exclusion (Fig. 2), demonstrating a solid non-heterogeneous construct. The VAS also presented good internal reliability (α = 0.81). The parosmia subscale showed acceptable results (α = 0.75), while the sincerity statement presented moderate to low levels of reliability (α = 0.5). Accordingly, the internal consistency values in the sincerity statement were importantly heterogeneous (range 0.37‒0.54), similar in both centers (α = 0.42, Londrina; α = 0.58, Salvador) (Table 2). Item-by-item consistency analyses are described in Table S3 and Fig. 2.

**Table 2:** Internal consistency with Cronbach's alpha and Cronbach's alpha variation if the item is excluded.

QOD statement	Cronbach α
Parosmia	0.76 (0.64‒0.71)
Quality of Life	0.86 (0.85‒0.89)
Sincerity	0.50 (0.37‒0.54)
VAS	0.82 (0.72‒0.82)

The data were described in median (IQR). VAS, Visual Analog Scale.

**Fig. 3:**
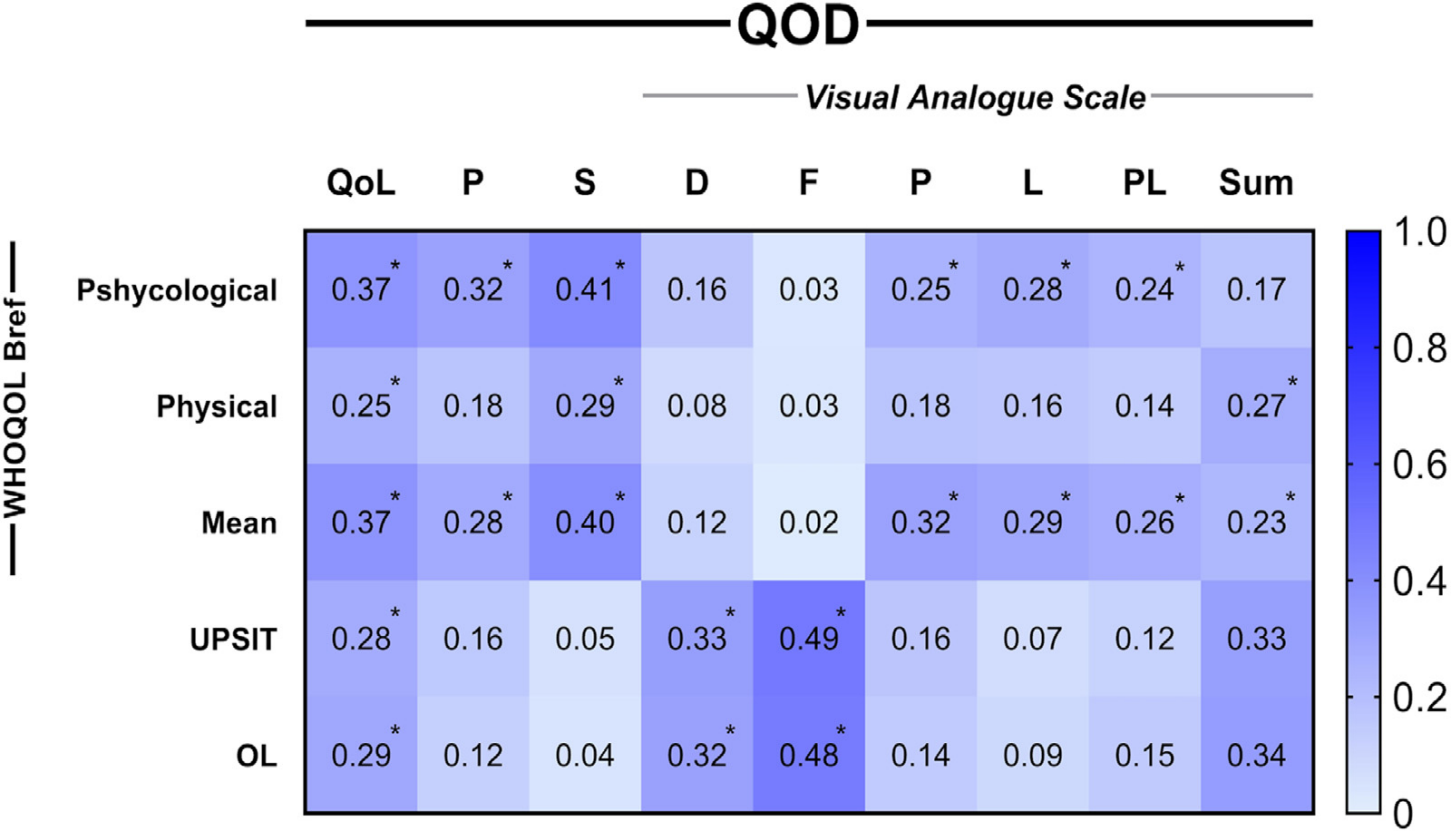
Size effect of content correlations between QOD domains and external content instruments. All spearman correlation coefficients are presented as module. Descriptive values can be found at supplementary material. QoL, Quality of Life; P, Parosmia; S, Sincerity; VAS: D, Disturbance; F, Frequency; P, Professional; L, Leisure; PL, Private Life; Sum, Summed scores of all VAS domains; OL, Olfactory Loss degrees (Mild, moderate and severe hyposmia and anosmia). *p < 0.05.

The content correlation was performed using the Spearman correlation test, identifying a significant correlation between the QOD-QoL domain and the UPSIT® raw score (Spearman's ρ = −0.28, *p* = 0.002) and degree of olfactory loss (ρ = 0.29, *p* = 0.001), indicating worse QoL associated with olfactory impairment (Fig. 3). There was a moderate correlation between the quality-of-life domain and the WHOQOL-bref general score (ρ = −0.37, *p* < 0.001). QOD-QoL also significantly correlated with WHOQOL-bref physical, psychological, social, and environment domains (ρ = −0.25, *p* = 0.006; ρ = −0.37, *p* < 0.001; ρ = −0.29, *p* = 0.001; ρ = −0.35, *p* = 0.000; respectively) (Table S4). QOD parosmia and VAS domains demonstrated significant association with WHOQOL-bref (ρ = −0.28, *p* = 0.001 and ρ = −0.23, *p* = 0.01, respectively) (Table S4). Split-half reliability test demonstrated stability in VAS, QoL and parosmia domains (0.903, 0.887 and 0.794 respectively), however, sincerity showed poor results (0.289) (Table S5).

The intra-questionnaire evaluation showed a good correlation between parosmia and QoL domains(ρ = 0.52, *p* < 0.001) (Table S4), indicating addictive QoL impairment when patients present with parosmia associated with dysosmia to olfactory quantitative dysfunction.

For test-retest validity, twelve patients (12/126, 9.5 %) had questionnaire re-application 4.1 (3.2‒5.3) months after the first visit. Patients were predominantly male (*n* = 8, 66.7 %), 32.3 (SD) years mean, with similar QOD mean, degree of olfactory alteration and olfactory loss etiology. A significant high effect size correlation was found between QoL and P test-retest analysis (*τ* = 0.89, *p* < 0.001; 0.837, *p* < 0.001, respectively) despite the long application time. Sincerity and VAS presented high levels of dispersion with no significant correlation between application, consonant to the split halves cross-sectional analysis. Additional correlation and descriptive information can be found in supplementary Tables S6 and S7.

## Discussion

The Olfactory Disorder Questionnaire (QOD) developed for Brazilian Portuguese speakers proved to be consistent and reliable, with satisfactory results to determine the impact of olfactory loss on quality of life. The present study included participants from two major centers in distinct regions of the country (South and Northeast), which ensured diverse cultural and linguistic representativeness in this sample. Different etiologies, educational levels, and clinical spectrum were considered in the validation analyses, testing different application backgrounds for the questionnaire.

The Questionnaire of Olfactory Dysfunction has achieved international recognition as a widely used QoL assessment tool, undergoing validation in various languages. The Chinese, Persian, and Korean validations achieved similar results with 104, 113, and 213 patient evaluations, respectively, using similar methodologies. Cronbach alpha ranged from 0.814‒0.909 in these studies, Korean and Persian studies found low confidence in the sincerity domain (α = 0.243 and 2.50, respectively), while the Chinese cohort presented insufficient metrics in the parosmia domain evaluation (α = 0.473).^25^, ^26^, ^27^ However, other validations used abridged versions of QOD, such as the Greek validation[28] for QoL domain validation (Cronbach's α of 0.91) and the Spanish validation (Cronbach's α of QOD-QoL-Negative Statements 0.861).^29^ Additionally, in the English validation published in 2019, the modified questionnaire was used to create an online version of QOD (e-ODQ), whose Cronbach's α for internal consistency was 0.888 for QOD. During the SARS-CoV-2 pandemic, the e-ODQ was used for Portugal QOD validation. The European QOD-pt had 110 participants who completed the modified e-ODQ and the Sniffin’ Sticks Test (Cronbach's α for internal consistency of QOD 0.924)[30] and validated the questionnaire for Portuguese in Portugal, which is grammatically, semantically, and culturally different from Brazilian Portuguese.

The internal consistency of the QoL statement in the studied cohort falls within optimal limits (Cronbach's alpha of QOD-QOL = 0.86), as observed in other validation studies of this questionnaire.^25^, ^26^, ^27^, ^28^, ^29^, ^30^, ^31^ The sincerity statement (QOD-S) did not show a statistically significant correlation with either the raw score of UPSIT® (τ = 0.054; *p* = 0.5) or the degree of olfactory loss (τ = −0.034; *p* = 0.70). Additionally, this domain had a much lower level of reliability than expected (Cronbach's alpha QOD-S 0.505). These results were also observed in other publications that sought to validate the questionnaire.^26^^,^^27^

Prior studies encountered similar findings regarding low internal consistency coefficients, possibly indicating intrinsic domain fragilities.^26^^,^^27^ The Persian version of QOD found unsatisfactory internal consistency in the sincerity domain does not affect the overall reliability of the questionnaire, and the study showed that QOD has reliability if the S statement is omitted, achieving a Cronbach's alpha of 0.89. The consistency of the present findings with current literature reinforces the possibility the sincerity domain has intrinsic problems, possibly associated with excessive subjectivity, difficult cultural adaptation, and non-specific clinical value. Fortunately, QOD domains are interpreted independently, and this statement does not seem to hinder the assessment of quality of life.^27^ Future reevaluation of this domain may be necessary.

Regarding the parosmia statement, the present results showed satisfactory results (Cronbach's alpha of QOD-P 0.76). This finding is compatible with the Persian and Korean versions of QOD,^26^^,^^27^ though was not observed in the validation of QOD for the Chinese population, which showed low internal reliability in this statement (Cronbach's alpha of QOD-P 0.47).^25^ It is important to emphasize that Cronbach's alpha may be limited as it analyzes a few items from the instruments, possibly underestimating coefficient metrics. Therefore, the parosmia statement being a 4-item domain is susceptible to this type of bias. The authors of the validations recommend some modifications in the parosmia statement for a better evaluation of this statement.^25^^,^^27^

Unlike prior studies, the validation study was performed in a multi-center setting with significant sample differences, allowing for a more comprehensive evaluation in a diverse population with varied education and etiologies of olfactory dysfunction. Collection centers differed in their specialty and sector: the South center was located in a private sector clinic while the Northeast center is part of the public healthcare system. Despite the differences in centers and populations served, both cohorts presented similar and statistically significant results strengthening the validation of the QOD for Brazil.

Some limitations of the present study should be highlighted. The long time between the first application and the retest is considered a limitation, considering that other studies established a period of two weeks between the applications.^25^^,^^32^ Nevertheless, it is noteworthy that other authors have chosen to maintain an unrestricted time frame, specifying only a minimum interval between the initial test and the retest.^28^ Moreover, a limited number of patients responded to the request to visit the study center for the test's reapplication. The authors attribute this, in part, to the challenging circumstances imposed by the ongoing pandemic. Additionally, some participants may experience exhaustion in the process due to the administration of multiple questionnaires (WHOQOL-bref, UPSIT®, and QOD).

## Conclusions

The findings of the present study demonstrate the successful validation of the QOD in its adapted version, yielding highly satisfactory results. This represents a notable breakthrough, as it establishes the QOD as a dependable clinical and scientific instrument that can be effectively employed within the Brazilian population.

## Declaration of competing interest

The authors declare no conflicts of interest.

The authors have no personal or financial affiliations that could compromise the objectivity or integrity of the information presented in this study. This article follows the STROBE statement (Strengthening the Reporting of Observational Studies in Epidemiology).

## Appendix Supplementary materials

Image, application 1

## References

[1] Murphy C., Schubert C.R., Cruickshanks K.J., Klein B.E., Klein R., Nondahl D.M. (2002). Prevalence of olfactory impairment in older adults. JAMA.

[2] Sivam A., Wroblewski K.E., Alkorta-Aranburu G., Barnes L.L., Wilson R.S., Bennett D.A. (2016). Olfactory dysfunction in older adults is associated with feelings of depression and loneliness. Chem Senses.

[3] Siegel J.K., Kung S.Y., Wroblewski K.E., Kern D.W., McClintock M.K., Pinto J.M. (2021). Olfaction is associated with sexual motivation and satisfaction in older men and women. J Sex Med.

[4] Liljas A.E.M., Jones A., Cadar D., Steptoe A., Lassale C. (2020). Association of multisensory impairment with quality of life and depression in english older adults. JAMA Otolaryngol Head Neck Surg.

[5] Hummel T., Whitcroft K.L., Andrews P., Altundag A., Cinghi C., Costanzo R.M. (2016). Position paper on olfactory dysfunction. Rhinology.

[6] Ciofalo A., Filiaci F., Romeo R., Zambetti G., Vestri A.R (2006). Epidemiological aspects of olfactory dysfunction. Rhinology.

[7] Fark T., Hummel T. (2013). Olfactory disorders: distribution according to age and gender in 3,400 patients. Eur Arch Otorhinolaryngol.

[8] Sánchez-Vallecillo M.V., Fraire M.E., Baena-Cagnani C., Zernotti M.E. (2012). Olfactory dysfunction in patients with chronic rhinosinusitis. Int J Otolaryngol.

[9] Schriever V.A., Studt F., Smitka M., Grosser K., Hummel T. (2014). Olfactory function after mild head injury in children. Chem Senses.

[10] Vennemann M.M., Hummel T., Berger K. (2008). The association between smoking and smell and taste impairment in the general population. J Neurol.

[11] Duncan H.J., Seiden A.M. (1995). Long-term follow-up of olfactory loss secondary to head trauma and upper respiratory tract infection. Arch Otolaryngol Head Neck Surg.

[12] Politis M., Wu K., Molloy S., Bain P.G., Chaudhuri K.R., Piccini P. (2010). Parkinson's disease symptoms: the patient's perspective. Mov Disord.

[13] Roberts R.O., Christianson T.J., Kremers W.K., Mielke M.M., Machulda M.M., Vassilaki M. (2016). Association between olfactory dysfunction and amnestic mild cognitive impairment and alzheimer disease dementia. JAMA Neurol.

[14] Huang C., Huang L., Wang Y., Li X., Ren L., Gu X. (2021). 6-month consequences of COVID-19 in patients discharged from hospital: a cohort study. Lancet.

[15] Gregorio L.L., Caparroz F., Nunes L.M., Neves L.R., Macoto E.K. (2014). Olfaction disorders: retrospective study. Braz J Otorhinolaryngol.

[16] (1998). Development of the World Health Organization WHOQOL-BREF quality of life assessment. The WHOQOL group. Psychol Med.

[17] Cohen S.R., Mount B.M., Strobel M.G., Bui F. (1995). The McGill Quality of Life Questionnaire: a measure of quality of life appropriate for people with advanced disease. A preliminary study of validity and acceptability. Palliat Med.

[18] Ware J.E., Sherbourne C.D (1992). The MOS 36-item short-form health survey (SF-36). I. conceptual framework and item selection. Med Care.

[19] Kollndorfer K., Reichert J.L., Brückler B., Hinterleitner V., Schöpf V. (2017). Self-esteem as an important factor in quality of life and depressive symptoms in anosmia: a pilot study. Clin Otolaryngol.

[20] Frasnelli J., Hummel T. (2005). Olfactory dysfunction and daily life. Eur Arch Otorhinolaryngol.

[21] Doty R.L., Shaman P., Dann M. (1984). Development of the University of Pennsylvania Smell Identification Test: a standardized microencapsulated test of olfactory function. Physiol Behav.

[22] Beaton D.E., Bombardier C., Guillemin F., Ferraz M.B. (2000). Guidelines for the process of cross-cultural adaptation of self-report measures. Spine.

[23] Smeets M.A.M., Veldhuizen M.G., Galle S., Goudelock J., de Haan A.J.A., Vernooij J. (2009). Sense of smell disorder and health-related quality of life. Rehabil Psychol.

[24] Fornazieri M.A., Doty R.L., Santos C.A., Pinna F.R., Bezerra T.F., Voegels R.L. (2013). A new cultural adaptation of the university of pennsylvania smell identification test. Clinics (Sao Paulo).

[25] Yang D., Wang J., Ni D., Liu J., Wang X. (2016). Reliability and validity of the Chinese version of the questionnaire of olfactory disorders (QOD) when used with patients having olfactory dysfunction. Eur Arch Otorhinolaryngol.

[26] Choi W.R., Jeong H.Y., Kim J.H. (2018). Reliability and validity of the Korean version of the questionnaire of olfactory disorders. Int Forum Allergy Rhinol.

[27] Jalessi M., Kamrava S.K., Amini E., Rafiei F., Nasouti M.A., Moosavi N. (2017). Is the persian version of the “olfactory disorder questionnaire” reliable and valid?. Iran J Otorhinolaryngol.

[28] Simopoulos E., Katotomichelakis M., Gouveris H., Tripsianis G., Livaditis M., Danielides V. (2012). Olfaction-associated quality of life in chronic rhinosinusitis: adaptation and validation of an olfaction-specific questionnaire. Laryngoscope.

[29] Chiesa-Estomba C.M., Lechien J.R., Calvo-Henríquez C., Mayo M., Maldonado B., Maza J. (2021). Translation and validation of the short version of the Questionnaire of Olfactory Disorders-Negative Statements to Spanish. Am J Otolaryngol.

[30] De Sousa Machado A., Sousa F., Costa J., Silva A., Pinto A., Simmen D. (2022). Adaptation and validation of portuguese version of olfactory disorders questionnaire (PT-ODQ). Cureus.

[31] Langstaff L., Pradhan N., Clark A., Boak D., Salam M., Hummel T. (2019). Validation of the olfactory disorders questionnaire for English-speaking patients with olfactory disorders. Clin Otolaryngol.

[32] Pirola F., Giombi F., Ferreli F., Costantino A., Mercante G., Paoletti G. (2022). Cross-cultural validation of the short version of the questionnaire of olfactory disorders-negative statements into Italian: towards personalized patient care. J Pers Med.

