# Supplementary material for: Validation of the questionnaire of olfactory disorders (QOD) for the Brazilian population
Source: Clinics (Sao Paulo). 2024 Jun 14;79:100414. doi: 10.1016/j.clinsp.2024.100414 (PMC11226810; doi:10.1016/j.clinsp.2024.100414)

**CLINICS-D-23-00407_Supplementary Material**

**Table S1** Original Version of the QOD in English.

| **Item** | **Questions** | | | **Answers** | | | |
| --- | --- | --- | --- | --- | --- | --- | --- |
|  |  |  |  | **I agree** | **I agree partly** | **I disagree partly** | **I disagree** |
| P1 | Food tastes different from what used to. | | |  |  |  |  |
| P2 | Often I perceive a bad smell, regardless whether a potential odor source is present. | | |  |  |  |  |
| P3 | Other people find odors pleasant which are unpleasant to me. | | |  |  |  |  |
| P5 | My biggest problem is not that odors are less intense (or absent), but that things smell different from what they used to. | | |  |  |  |  |
| 1 | Because of my smell disorder, I go to restaurants less often than I used to. | | |  |  |  |  |
| 4 | From waking up until bedtime, I am aware of my difficulties with smelling. | | |  |  |  |  |
| 11 | The difficulties with smelling impair my appreciation of drinks and foods. | | |  |  |  |  |
| 13 | I am worrying if I will never be able to handle this problem. | | |  |  |  |  |
| 14 | I always keep a promise, no matter how difficult it is to do what I have promised. | | |  |  |  |  |
| 15 | Because of the changes in my smelling ability, I feel more tense than I used to be. | | |  |  |  |  |
| 17 | Sometimes I have thoughts and ideas I would not want other people to know of. | | |  |  |  |  |
| 19 | Most of my problems are due to the difficulties with smelling. | | |  |  |  |  |
| 22 | The difficulties with smelling disturb me when I am eating | | |  |  |  |  |
| 23 | My behavior is always good and impeccable. | | |  |  |  |  |
| 26 | Because of my difficulties with smelling, I visit friends, relatives or neighbors less often. | | |  |  |  |  |
| 27 | Because of my difficulties with smelling, I try harder to relax. | | |  |  |  |  |
| 28 | Because of my difficulties with smelling, I have weight problems | | |  |  |  |  |
| 31 | Among all the people I know, there are some I cannot stand at all. | | |  |  |  |  |
| 32 | I can imagine to adjust to my difficulties with smelling. | | |  |  |  |  |
| 33 | The difficulties with smelling make me feel being ostracized. | | |  |  |  |  |
| 34 | Because of my problems with smelling I avoid groups of people. | | |  |  |  |  |
| 35 | Difficulties with smelling are among the problems of life, one has to live with. | | |  |  |  |  |
| 36 | I never ever was late at an appointment or at work. | | |  |  |  |  |
| 37 | Because of my problems with smelling I eat more\less than in the past. | | |  |  |  |  |
| 39 | Because of the difficulties with smelling, I am scared of getting exposed to certain dangers (e.g, gas, rotten food). | | |  |  |  |  |
| 42 | Because of the difficulties with smelling, I have problems with taking part in the activities of the daily life. | | |  |  |  |  |
| 48 | Sometimes I am talking of things I do not understand. | | |  |  |  |  |
| 49 | The difficulties with smelling make me feel angry | | |  |  |  |  |
| 50 | Because of the difficulties with smelling, my relationship to my spouse is disturbed. | | |  |  |  |  |
| Please use the scale below to rate how annoying the difficulties with smelling are to you. | | | | | | | |
| Not annoying at all | |  | Extremely annoying | | | |  |
| Please use the scale below to rate how often you become aware of the difficulties with smelling. | | | | | | | |
| Never | |  | Extremely | | | |  |
| Please indicate on the scale below how severely the difficulties with smelling affected your professional performance during the last month. | | | | | | | |
| Not at all | |  | Extremely | | | |  |
| Please indicate on the scale below how severely the difficulties with smelling affected your recreational activities during the last month. | | | | | | | |
| Not at all | |  | Extremely | | | |  |
| Please indicate on the scale below how severely the difficulties with smelling affected your private life during the last month. | | | | | | | |
| Not at all | |  | Extremely | | | |  |

**Table S2** Final Portuguese version of the Questionnaire of Olfactory Disorders.

| **Item** | **Questões** | **Respostas** | | | |
| --- | --- | --- | --- | --- | --- |
|  |  | **Eu concordo** | **Eu concordo parcialmente** | **Eu discordo parcialmente** | **Eu discordo** |
| P1 | Os gostos dos alimentos estão diferentes do que costumavam ser. |  |  |  |  |
| P2 | Frequentemente, percebo um mal cheiro, independente da presença de uma fonte de odor em potencial. |  |  |  |  |
| P3 | Outras pessoas caracterizam como agradáveis certos odores que são desagradáveis para mim. |  |  |  |  |
| P4 | Meu maior problema não é que os odores são menos intensos ou ausentes, mas que algumas coisas cheiram diferentes do que costumavam. |  |  |  |  |
| 1 | Por causa do meu problema com o olfato, eu vou a restaurantes com menor frequência do que antigamente. |  |  |  |  |
| 4 | Desde o momento em que acordo até a hora de dormir, estou consciente das minhas dificuldades de sentir odores. |  |  |  |  |
| 11 | As dificuldades olfativas prejudicam minha apreciação de bebidas e comidas. |  |  |  |  |
| 13 | Estou preocupado se jamais serei capaz de lidar com esse problema. |  |  |  |  |
| 14 | Eu sempre mantenho uma promessa, não importa o quão difícil seja realizar o que prometi. |  |  |  |  |
| 15 | Devido às mudanças na minha habilidade olfativa, sinto-me mais tenso do que habituava ser. |  |  |  |  |
| 17 | Às vezes tenho ideias e pensamentos que não gostaria que os outros soubessem. |  |  |  |  |
| 19 | A maioria dos meus problemas se devem as minhas dificuldades com odores . |  |  |  |  |
| 22 | Os problemas com odores me perturbam enquanto estou comendo. |  |  |  |  |
| 23 | Meu comportamento é sempre bom e impecável. |  |  |  |  |
| 26 | Devido às dificuldades com cheiros, visito amigos, parentes e vizinhos com menor frequência. |  |  |  |  |
| 27 | Devido às dificuldades olfatórias, esforço-me mais para conseguir relaxar. |  |  |  |  |
| 28 | Devido às dificuldades com cheiros, tenho problemas com meu peso. |  |  |  |  |
| 31 | Entre todas as pessoas que conheço, há algumas às quais não consigo permanecer ao lado. |  |  |  |  |
| 32 | Eu consigo usar a imaginação para ajustar-me às minhas dificuldades olfatórias. |  |  |  |  |
| 33 | Os problemas com o olfato fazem-me sentir marginalizado/isolado. |  |  |  |  |
| 34 | Devido às dificuldades com odores, eu evito grupos de pessoas. |  |  |  |  |
| 35 | Dificuldades com odores estão entre os problemas da vida, com um dos quais tem-se de conviver |  |  |  |  |
| 36 | Eu nunca estou atrasado para um compromisso ou para o trabalho. |  |  |  |  |
| 37 | Devido aos meus problemas com cheiros, consumo mais/menos alimentos do que antigamente. |  |  |  |  |
| 39 | Devido às dificuldades com odores, tenho medo de ficar exposto a certos perigos (gás, comida podre, etc.). |  |  |  |  |
| 42 | Devido a minha dificuldade com odores, tenho problemas para realizar atividades do dia a dia. |  |  |  |  |
| 48 | Às vezes, estou falando de coisas que não entendo. |  |  |  |  |
| 49 | Meus problemas com cheiros deixam-me zangado. |  |  |  |  |
| 50 | Devido aos problemas com odores, o relacionamento com meu cônjuge encontra-se perturbado. |  |  |  |  |

Por favor, use a escala abaixo para avaliar o quão **incômodas** as dificuldades com odores são para você.

Nada Incomodado                                                                                           Extremamente incomodado


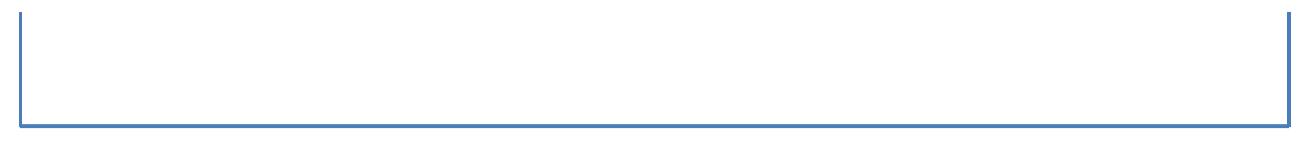


Por favor, use a escala abaixo para avaliar com que **frequência** torna-se ciente/ se preocupa com seu distúrbio olfatório.

Nunca                                                                                                                                              Sempre


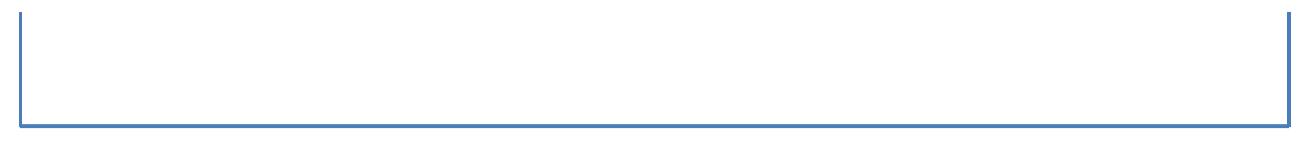


Por favor, indique na escala abaixo o quão severamente seu problema olfatório afetou sua **atuação profissional** no último mês.

Não afetou                                                                                                                Afetou extremamente


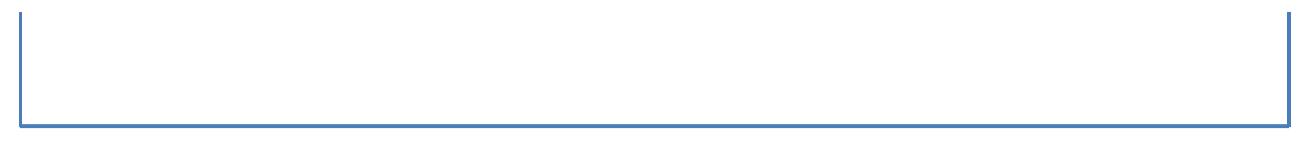


Por favor, indique na escala abaixo o quão severamente seu distúrbio com cheiros afetou suas **atividades recreativas** no mês passado.

Não afetou                                                                                                                Afetou extremamente


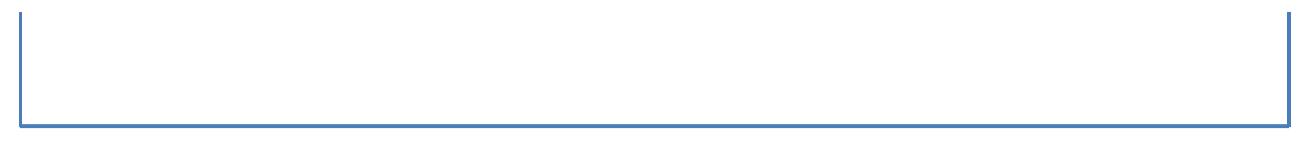


Por favor, indique na escala abaixo o quão severamente seu distúrbio olfatório afetou sua **vida privada** no mês passado.

Não Afetou                                                                                                              Afetou Extremamente


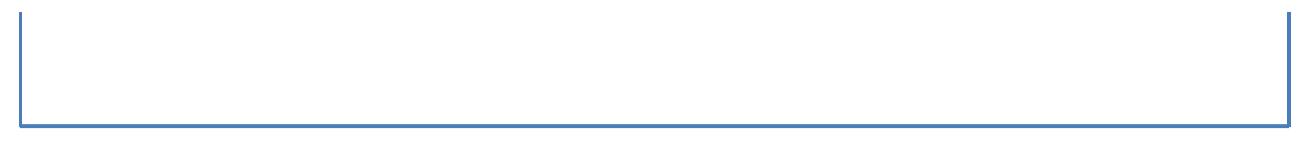


**Table S3** Analysis of Cronbach's alpha if each item is excluded for QOD quality of life and sincerity domains.

| **Scale item** | **Cronbach’s alfa if the item is excluded** |
| --- | --- |
| Quality of Life |  |
| Q5 | 0.850 |
| Q6 | 0.852 |
| Q7 | 0.851 |
| Q8 | 0.845 |
| Q10 | 0.846 |
| Q12 | 0.853 |
| Q13 | 0.848 |
| Q15 | 0.856 |
| Q16 | 0.853 |
| Q17 | 0.856 |
| Q19 | 0.883 |
| Q20 | 0.860 |
| Q21 | 0.858 |
| Q22 | 0.886 |
| Q24 | 0.857 |
| Q25 | 0.854 |
| Q26 | 0.845 |
| Q28 | 0.855 |
| Q29 | 0.850 |
| Sincerity |  |
| Q9 | 0.485 |
| Q11 | 0.410 |
| Q14 | 0.451 |
| Q18 | 0.370 |
| Q23 | 0.538 |
| Q27 | 0.478 |

**Table S4** Size of effect of content correlations between QOD domains, WHOQOL-Bref and UPSIT questionnaires. Statistically significant correlations in spearman correlation test are highlighted in orange.

|  |  |  |  |  | **VISUAL ANALOGUE SCALE (VAS)** | | | | |
| --- | --- | --- | --- | --- | --- | --- | --- | --- | --- |
|  | **Variables** | **QOD-QOL** | **QOD-P** | **QOD-S** | **Disturbance** | **Frequency** | **Profissional** | **Leisure** | **Private Life** |
| **QOD** | QOD-QOL | 1,000 |  |  |  |  |  |  |  |
|  | QOD-P | 0,515 | 1,000 |  |  |  |  |  |  |
|  | QOD-S | 0,202 | 0,243 | 1,000 |  |  |  |  |  |
|  | EVA disturbance | 0,735 | 0,361 | 0,144 | 1,000 |  |  |  |  |
|  | EVA frequency | 0,598 | 0,363 | 0,053 | 0,694 | 1,000 |  |  |  |
|  | EVA professional | 0,320 | 0,267 | 0,118 | 0,192 | 0,212 | 1,000 |  |  |
|  | EVA leisure | 0,590 | 0,258 | 0,303 | 0,428 | 0,320 | 0,372 | 1,000 |  |
|  | EVA private life | 0,562 | 0,147 | 0,214 | 0,451 | 0,325 | 0,357 | 0,757 | 1,000 |
|  |  |  |  |  |  |  |  |  |  |
| **WHOQOL – Bref** | WHOQOL mean | -0,374 | -0,280 | -0,400 | -0,116 | -0,015 | -0,316 | -0,293 | -0,261 |
|  | WHOQOL physical | -0,246 | -0,175 | -0,284 | -0,082 | -0,031 | -0,178 | -0,162 | -0,143 |
|  | WHOQOL mental | -0,367 | -0,314 | -0,412 | -0,164 | -0,026 | -0,247 | -0,280 | -0,238 |
|  | WHOQOL social | -0,287 | -0,218 | -0,314 | -0,071 | -0,019 | -0,326 | -0,265 | -0,272 |
|  | WHOQOL environment | -0,348 | -0,216 | -0,288 | -0,066 | -0,029 | -0,386 | -0,320 | -0,254 |
|  |  |  |  |  |  |  |  |  |  |
| **UPSIT** | UPSIT | -0,275 | -0,155 | 0,054 | -0,331 | -0,485 | -0,164 | -0,069 | -0,116 |
|  | Degree of olfactory loss | 0,291 | 0,124 | -0,340 | 0,323 | 0,475 | 0,144 | 0,090 | 0,150 |

**Table S5** Consistency coeficients stratfied by QOD domain. Split-half test was acessed using odd-even item segregation.

| **QOD Statement** | **Cronbach Alpha** | **Split-Half** |
| --- | --- | --- |
| Parosmia | 0.760 (0.64‒0.71) | 0.794 |
| Quality of Life | 0.860 (0.84‒0.89) | 0.903 |
| Sincerity | 0.500 (0.37‒0.54) | 0.289 |
| Visual Analogue Scale | 0.820 (0.72‒0.82) | 0.887 |

**Table S6** Size effect correlation between Test-Retest and QOD statements and VAS.

| **Test Retest, N = 12** | **r coefficient** | **P value** |
| --- | --- | --- |
| **Life Quality** | 0,829 | <0,001 |
| **Parosmia** | 0,837 | <0,001 |
| **Sincerity** | 0,216 | 0,503 |
| **VAS Annoying** | 0,401 | 0,196 |
| **VAS Frequency** | 0,160 | 0,619 |
| **VAS Professional** | 0,125 | 0,699 |
| **VAS Free Time** | 0,516 | 0,086 |
| **VAS Private Life** | 0,537 | 0,072 |

**Table S7** Sociodemographic and clinical characteristics of the Test- Retest population.

| **Variable** | **Total**  **(n = 12)** |
| --- | --- |
| Age (years), mean | 32.3 |
| Female gender - n (%) | 4 (33.3) |
| **Time between applications in months - median (IQR)** | 4,06 (2,1) |
| **Etiologies -n (%)**  Post-infectious  Others (idiopathic and traumatic) | 9 (75)  3 (25) |
| **QOD, mean**  Sincerity  Quality of life  Parosmia | 7.2  20  6.2 |
| **UPSIT, mean (SD)**  Mild loss  Moderate loss  Severe loss  Anosmia | 18.7  1 (7.7)  1 (7.7)  5 (38.5)  5 (38.5) |

**Figure S1** Short version of the questionnaire developed by the World Health Organization Quality of Life Group (WHOQOL-bref) in Portuguese.


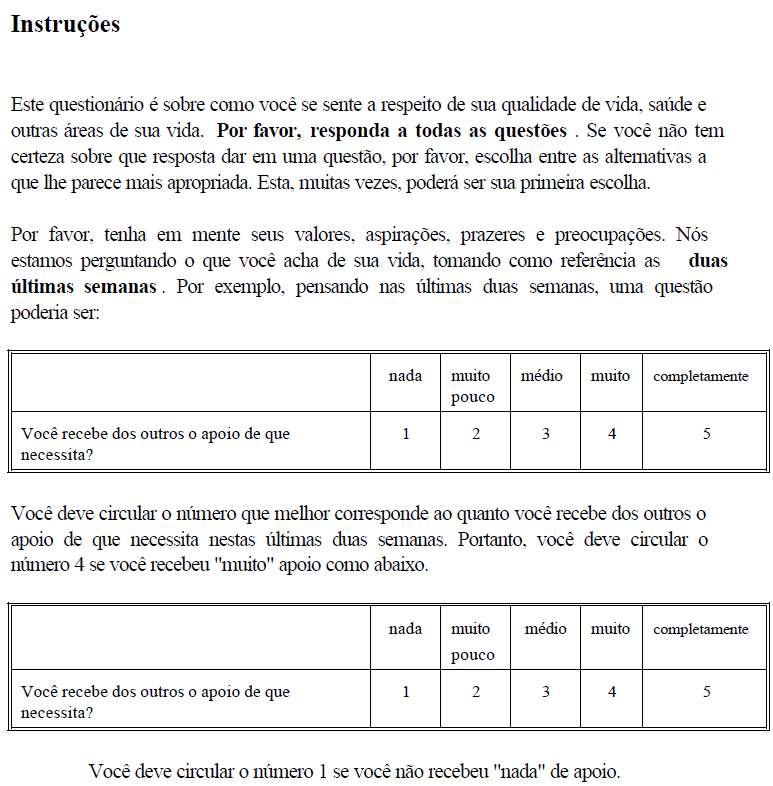


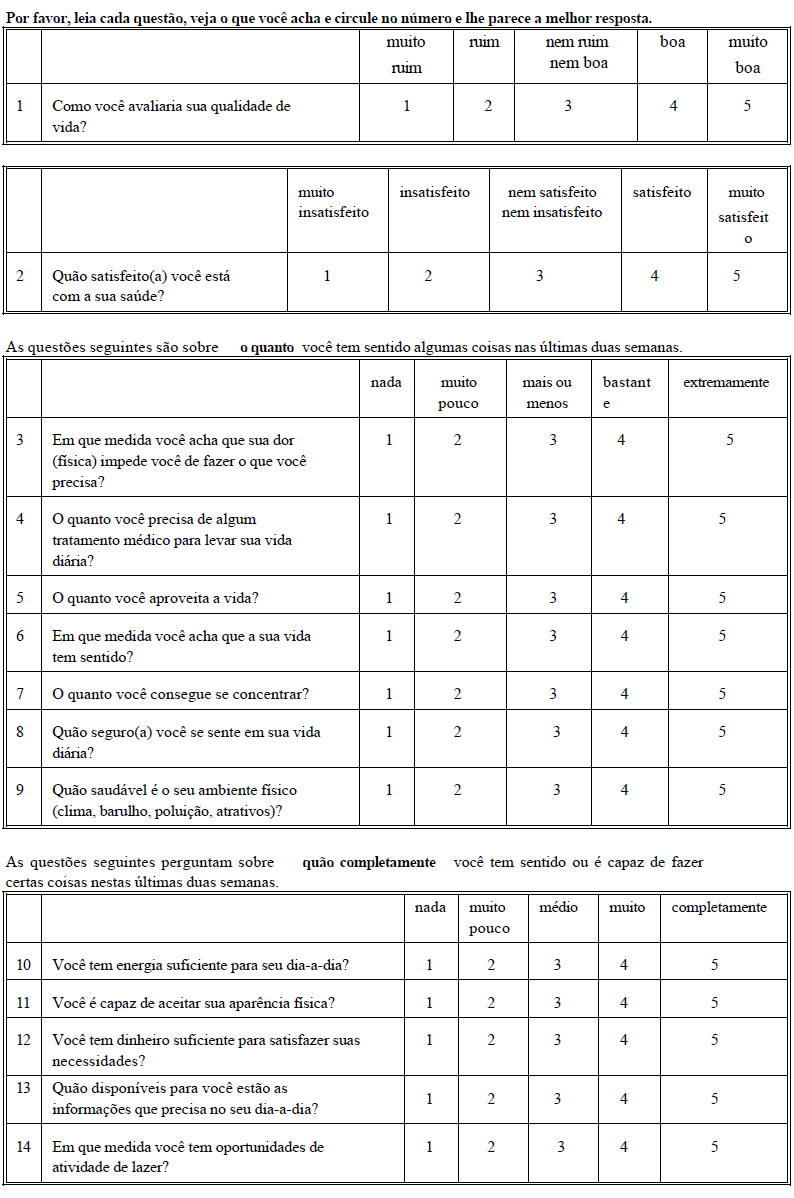

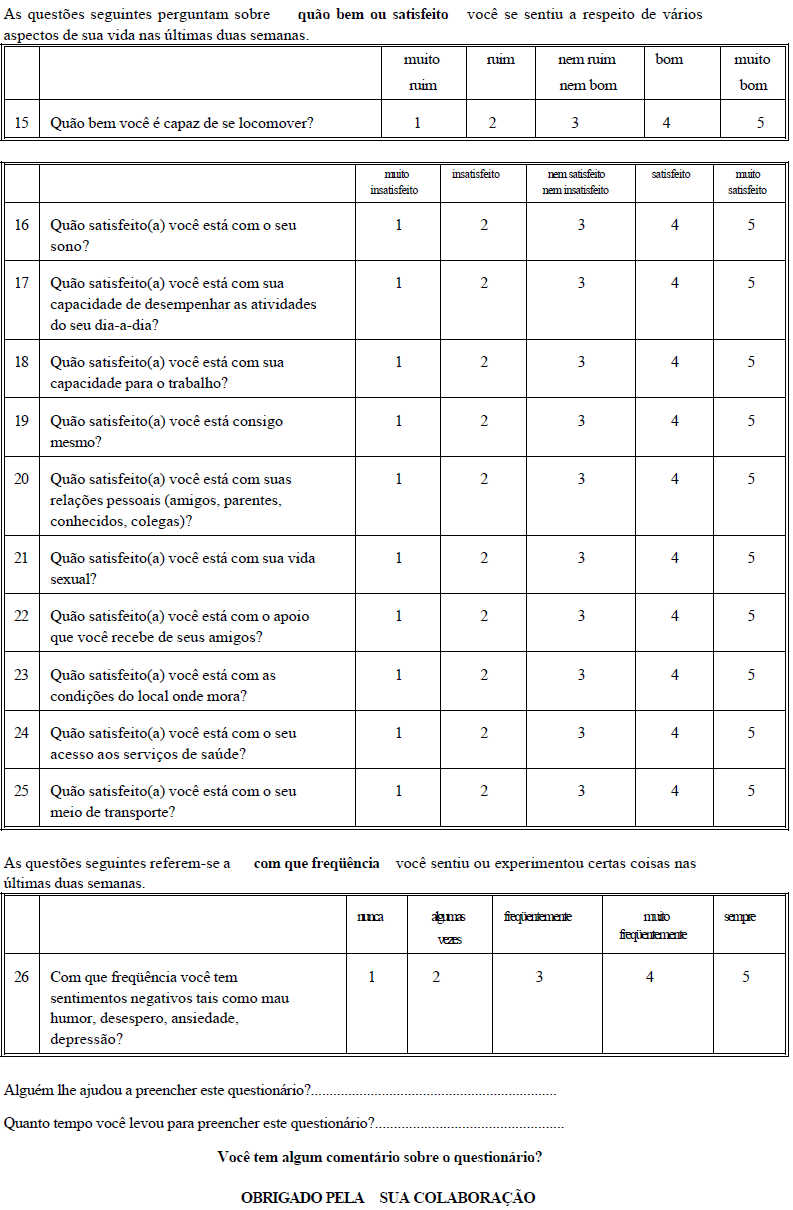

Supplement: Supplementary file 1 [file mmc1.docx]
